# Infection-osteogenesis crosstalk on TiO_2_ nanotube surfaces: mechanisms and synergistic design strategies

**DOI:** 10.3389/fbioe.2026.1926474

**Published:** 2026-10-29

**Authors:** Xiu Yang, Long-Can Wu, Hui-Dong Chen, Jie Xiao, Han-Lin Chen, Hui Wang, Jin-Shui Chen

**Affiliations:** Spinal Ward, The 900th Hospital of PLA Joint Logistic Support Force, Fuzong Clinical Medical College of Fujian Medical University, FuZhou, China

**Keywords:** antibacterial coating, immunomodulation, infection-osteogenesis crosstalk, macrophage polarization, osseointegration, smart responsive surface, TiO_2_ nanotubes, titanium implant

## Abstract

Titanium dioxide nanotubes (TNTs) fabricated via anodization have emerged as a versatile platform for surface modification of orthopedic and dental implants, owing to their tunable nanotopography and excellent potential for multifunctional biofunctionalization. However, the concurrent challenges of implant-associated bacterial infection and insufficient osseointegration remain formidable obstacles in clinical practice. Rather than being independent processes, bacterial infection and bone regeneration are intimately coupled through a complex signaling network—a concept increasingly recognized as the “infection-osteogenesis crosstalk.” We begin with how TNT morphology—nanotube diameter above all—differentially steers osteoblast behavior and bacterial adhesion, and how the initial protein corona and immune microenvironment set the infection-to-bone-healing trajectory. From signaling, we move to the pathways behind infection-driven osteogenic suppression: the core signaling pathways through which bacterial virulence factors suppress osteogenesis, including TLR-mediated NF-κB activation, RANKL/OPG imbalance-driven osteoclastogenesis, and biofilm hypoxia-induced metabolic reprogramming. We then look at how macrophages (M1/M2 polarization, NLRP3 inflammasome), neutrophils (NETosis), and T cells shape the host response to infection and bone repair. We close with current synergistic design strategies—metal ion doping, antimicrobial peptide loading, organic-inorganic composite coatings, temporally controlled drug release—and next-generation smart responsive surfaces including pH-responsive, photothermal/photocatalytic, enzyme-responsive, and immunomodulatory coatings. This review aims to provide a mechanistic framework for understanding infection-osteogenesis crosstalk on TNT surfaces and to guide the rational design of dual-functional implants that simultaneously combat infection and promote bone regeneration.

## 1 Introduction

Titanium and its alloys have long been the gold standard materials for orthopedic implants and dental fixtures due to their excellent mechanical properties, corrosion resistance, and biocompatibility (Kulkarni et al., 2015; Li et al., 2023; Yeo, 2019). Despite their widespread clinical success, titanium-based implants face two persistent challenges: implant-associated bacterial infections—particularly biofilm-mediated refractory infections caused by drug-resistant strains—and inadequate osseointegration at the bone-implant interface, which collectively contribute to implant failure and revision surgeries (Busscher et al., 2012; Arciola et al., 2018; Li et al., 2019c). The seminal concept of the “race for the surface” posits that the fate of an implant is determined by the competition between bacterial colonization and host tissue integration at its surface (Gristina, 1987; Busscher et al., 2012), a paradigm that has been experimentally validated in co-culture models (Subbiahdoss et al., 2009) and extended to the immune-evasive strategies of biofilms (Campoccia et al., 2019). With the aging global population and increasing volume of joint replacement and dental implantation procedures, addressing the seemingly opposing requirements of antibacterial activity and osteogenic promotion has become a central pursuit in biomaterials research.

TiO_2_ nanotubes (TNTs), self-organized nanotube arrays fabricated by electrochemical anodization of titanium, have garnered considerable attention as a surface modification strategy (Choi et al., 2015). The distinctive nanotopography of TNTs, with precisely controllable diameters (10–200 nm) and lengths (hundreds of nanometers to several micrometers), offers unique opportunities to differentially regulate cellular and bacterial responses. Studies have demonstrated that TNT diameter exerts a profound influence on osteoblast adhesion, proliferation, and differentiation through integrin-mediated mechanotransduction pathways involving Kindlin-2/integrin β1/YAP and F-actin/XBP1 signaling (Tang et al., 2013; Song et al., 2018; Chang et al., 2024; Deng et al., 2024). Concomitantly, TNT topography significantly impacts initial bacterial adhesion and biofilm formation, with small-diameter tubes (20–30 nm) exhibiting notable anti-adhesive properties (Anitha et al., 2015; Wu et al., 2022). This morphology-dependent dual regulation provides a unique physical cue for designing implant surfaces with both antibacterial and osteogenic capacity.

Critically, the relationship between antibacterial requirements and osteogenic demands is not merely a trade-off but reflects a deep biological coupling governed by a complex cellular-molecular crosstalk network (Qiao et al., 2020; Abdulhameed et al., 2022). On one hand, bacterial colonization triggers host immune responses through Toll-like receptor (TLR) signaling, generating a cascade of pro-inflammatory cytokines that directly suppress osteoblast differentiation while simultaneously promoting osteoclast activation via the RANKL-RANK axis (Arciola et al., 2018; Busscher et al., 2012). On the other hand, the implant surface itself induces a host immune response—the foreign body response that ultimately defines the biocompatibility of any implantable material (Ratner, 2016)——particularly the balance between M1 and M2 macrophage polarization—that fundamentally determines the trajectory of early bone healing (Sun S. J. et al., 2013; Fu et al., 2023). Thus, designing antibacterial and osteogenic functionalities as independent, siloed objectives is unlikely to achieve genuine clinical success.

The concept of “infection-osteogenesis crosstalk” has therefore been proposed as a systems-level framework for understanding the multidimensional, dynamic interactions among bacterial colonization, immune responses, and bone tissue regeneration within the confined biological space of the implant surface (Qi et al., 2021; Zhao et al., 2025). For instance, macrophages sense TNT nanotopography through nuclear mechanotransduction pathways that modulate their inflammatory phenotype (Fu et al., 2023), while antibacterial ions (e.g., Ag^+^, Cu^2+^, Zn^2+^) eluted from TNTs can modulate osteogenic differentiation through immunomodulatory effects on macrophages (Chen et al., 2020; Yao Y. et al., 2025). Deciphering this crosstalk network is essential for designing synergistic surface coatings that not only eradicate pathogens but actively orchestrate a pro-osteogenic immune microenvironment.

This review provides a comprehensive overview of progress in this field from three perspectives: first, the biological microenvironment of TNT surfaces and the differential cellular/bacterial responses to nanotopographical cues; second, the core molecular mechanisms of infection-osteogenesis crosstalk, encompassing bacterial product signaling, immune cell regulation, and inflammation-bone metabolism coupling; and third, current synergistic design strategies for dual-functional antibacterial-osteogenic coatings and the emerging frontier of smart responsive surfaces (Figure 1). It should be noted that the majority of evidence discussed herein is derived from *in vitro* assays and preclinical animal models; clinical data on TNT-based dual-functional coatings remain absent, and this represents an important limitation that is explicitly acknowledged.

**FIGURE 1:**
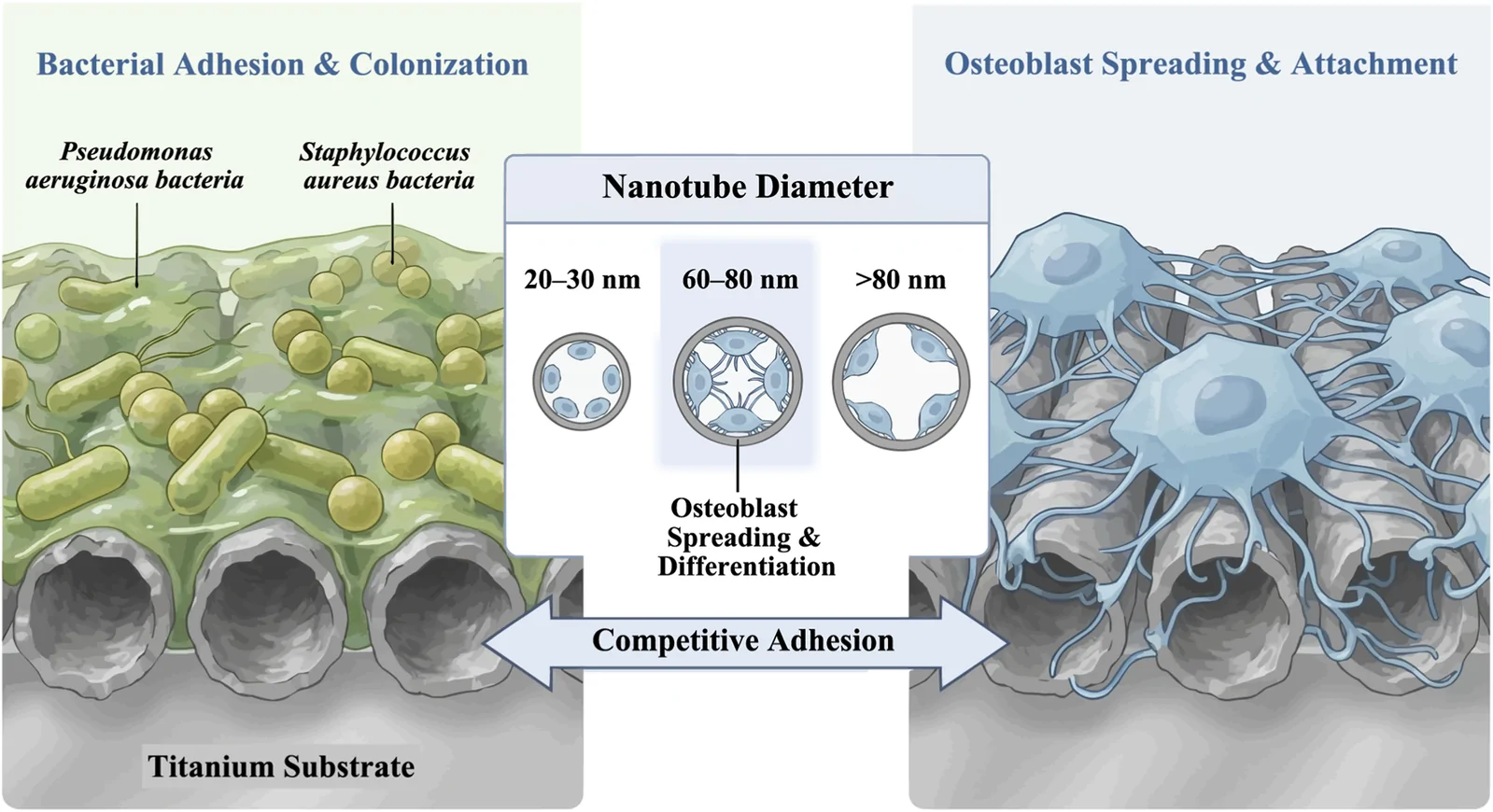
The “Race for the Surface” (Infection-Osteogenesis Crosstalk) on TiO_2_ Nanotubes. Schematic illustration depicting the competitive colonization between bacteria and osteoblasts on TiO_2_ nanotube (TNT) surfaces. Left panel: Bacterial adhesion and biofilm formation by *Staphylococcus aureus* and *Pseudomonas aeruginosa* on TNT surfaces. Right panel: Healthy osteoblast spreading and anchoring via abundant filopodia at nanotube apices. Center panel: Nanotube-diameter-dependent geometric effects on osteoblast filopodia grasping and bacterial adhesion, illustrating that the diameter range favoring early cell attachment and reduced bacterial colonization (∼20–30 nm) differs from that optimal for osteogenic differentiation (∼60–80 nm); the “best” diameter is therefore endpoint-dependent rather than a single universal optimum.

While numerous reviews have addressed TiO_2_ nanotubes (TNTs) from the perspectives of fabrication, drug delivery, or dual-functional surface modification (Lai et al., 2016; Li et al., 2019c; Wang et al., 2024), the present review distinguishes itself through a systems-level “infection-osteogenesis crosstalk” framework that explicitly recognizes bacterial infection and bone regeneration as biologically coupled processes rather than independent challenges. Compared with existing TNT reviews—which typically catalogue surface modifications or summarize antimicrobial and osteogenic data in parallel—our review offers three unique contributions: (i) a mechanistic integration of the core signaling pathways (TLR-NF-κB, RANKL/OPG, HIF-1α) through which bacterial virulence factors suppress osteogenesis, graded by evidence level; (ii) a comprehensive synthesis of the immune cell network—encompassing macrophage polarization, NLRP3 inflammasome activation, neutrophil NETosis, and T cell subset balance—as the central mediator of this crosstalk; and (iii) a systematic translation of these crosstalk mechanisms into rational design principles for next-generation smart responsive surfaces, together with an objective assessment of translational readiness. To our knowledge, no prior review has framed the TNT surface-biology interface through this integrated crosstalk lens, and we believe this conceptual integration, rather than the enumeration of individual studies, constitutes the principal novelty of the present work.

For the purpose of this review, we operationally define “infection-osteogenesis crosstalk” as the bidirectional and dynamic interplay among (i) bacterial colonization and biofilm formation on the implant surface, (ii) the host innate and adaptive immune response (macrophages, neutrophils, and T cells), and (iii) the osteogenic differentiation and bone remodeling programs of osteoblasts and osteoclasts, within the confined space of the bone–implant interface. The causal structure of this crosstalk comprises three principal arms: bacterial products activating inflammatory signaling (e.g., TLR–NF-κB) that directly suppresses osteogenesis; immune cells translating microbial stimuli into cytokine milieus that polarize bone-cell fate; and surface physicochemical cues that simultaneously modulate bacterial adhesion and osteogenic mechanotransduction. This operational definition provides the mechanistic backbone for the design principles discussed in Section 4, in which each engineering strategy is evaluated for its capacity to intervene in specific arms of this crosstalk.

## 2 Topographical regulation and biological microenvironment of TNT surfaces

### 2.1 Differential regulation of cellular and bacterial behavior by TNT morphological parameters

Nanotube diameter is the most critical topographical parameter governing the biological performance of TNT surfaces (Choi et al., 2015; Sun S. et al., 2013; Zhao et al., 2022). By precisely tuning the anodization voltage and electrolyte composition, TNT arrays with diameters ranging from 15 nm to over 200 nm can be reliably fabricated. The osteogenic response of bone-forming cells—including MC3T3-E1 pre-osteoblasts, MG63 osteoblast-like cells, and bone marrow mesenchymal stem cells (BMSCs)—exhibits a nonlinear dependence on tube diameter. Tubes of 15–30 nm diameter promote maximal cell adhesion and proliferation, while diameters of 60–80 nm elicit peak expression of osteogenic marker genes such as Runx2, Osterix, osteocalcin (OCN), and osteopontin (OPN). Beyond 100 nm, cell spreading area decreases and apoptosis increases (Sun S. et al., 2013; Xiong et al., 2026). Mechanistically, these effects are mediated by integrin-mediated focal adhesion kinase (FAK) signaling and YAP/TAZ nuclear translocation—TNT nanotopography modulates cytoskeletal tension, which in turn transduces mechanical signals through the Kindlin-2/integrin β1/YAP axis to promote osteogenic differentiation (Deng et al., 2024; Xiong et al., 2026). The involvement of filamentous actin (F-actin) rearrangement and XBP1 splicing in TNT-induced osteogenesis has also been documented (Chang et al., 2024).

The above findings reveal a critical and often overlooked divergence: the nanotube diameter that maximizes early cell adhesion and cytoskeletal organization (15–30 nm) is not the same as that which drives the highest level of osteogenic gene transcription (60–80 nm). This discrepancy is not contradictory but rather reflects the distinct mechanotransductive thresholds for different downstream events—integrin clustering and focal adhesion maturation require discrete nanoscale curvature, whereas osteogenic differentiation relies on more sustained cytoskeletal tension and YAP/TAZ nuclear translocation, which are optimally triggered on larger-diameter topographies (Chang et al., 2024; Deng et al., 2024).

Consequently, the widely cited “20–30 nm sweet spot” in the literature should be interpreted with caution. It primarily represents a compromise that favors early-stage anti-adhesive integration and moderate osteogenic priming, rather than a universally superior diameter for all aspects of bone regeneration. For applications prioritizing rapid bone matrix deposition, diameters in the 60–80 nm range may be more appropriate, provided that antibacterial strategies (e.g., drug loading or surface chemistry) are independently reinforced to compensate for their higher bacterial retention. Emerging hierarchical or gradient-diameter TNT designs offer a promising avenue to decouple these conflicting demands by presenting temporally or spatially varied topographical cues to sequentially guide bacterial exclusion, cell adhesion, and terminal differentiation.

In parallel, bacterial adhesion and biofilm formation on TNT surfaces exhibit a similar diameter dependence. Initial adhesion of clinically relevant pathogens such as *Staphylococcus aureus* and *Staphylococcus* epidermidis is significantly inhibited on TNTs with diameters of 20–30 nm, whereas bacterial retention increases on tubes exceeding 80 nm (Anitha et al., 2015; Wu et al., 2022). This selective antibacterial effect is attributed to two principal mechanisms: (i) the surface curvature of small-diameter TNTs (<30 nm) is incompatible with the contact geometry of bacterial pili and flagella, reducing attachment points; and (ii) the photocatalytic activity of anodized TiO_2_ generates reactive oxygen species (ROS) under ultraviolet-visible light excitation, causing oxidative damage to bacterial membranes (Lewandowska et al., 2015; Farrugia et al., 2021). Non-thermal plasma treatment has also been reported to effectively inhibit biofilm formation on TNT surfaces (Ji et al., 2023). Consequently, TNTs with diameters of approximately 20–30 nm have often been highlighted for their favorable balance between initial cell adhesion and reduced bacterial attachment. However, it is important to note that peak osteogenic differentiation, as reflected by Runx2 and osteocalcin expression, is consistently observed on larger-diameter tubes (∼60–80 nm), indicating that the “optimal” diameter is phase-dependent and should not be generalized without specifying the biological endpoint (Li et al., 2023). Beyond diameter, tube length and wall morphology also influence biological responses. Longer nanotubes (>1 μm) offer greater drug-loading capacity, although excessive aspect ratios may compromise mechanical integrity (MonchupaKingsak, 2022; Liu et al., 2021; Chen et al., 2026). Emerging topological designs, including asymmetric wall structures and gradient diameters, are being explored to further optimize mechanical-biological synergy. Composite TNT-containing materials, such as glass ionomer cements, have also demonstrated reduced aluminum release and improved cytocompatibility in dental applications (Morais et al., 2022).

### 2.2 Protein corona formation and the immune microenvironment as early gatekeepers of infection-osteogenesis balance

Upon implantation, the first biological events at the TNT surface are not direct cell or bacterial contacts but the rapid adsorption of proteins from bodily fluids, forming a “protein corona” (Morais et al., 2022; Qiao et al., 2020). The composition of this corona—including fibrinogen, albumin, immunoglobulins, and complement proteins—is highly dependent on the surface energy, wettability, and topography of the TNTs. The protein corona, in turn, dictates how arriving cells (immune cells, osteoprogenitors) and bacteria “perceive” the implant surface (Wu et al., 2021; Wang et al., 2023; Smith et al., 2010).

In the initial phase of infection-osteogenesis crosstalk, the protein corona exerts a decisive influence on macrophage polarization. TNT topographical features can directly modulate the conformation of adsorbed fibrinogen; based on established immunology, this may activate the Dectin-1–Syk–Card9 signaling axis in macrophages via integrin Mac-1 (CD11b/CD18) and contribute to a pro-inflammatory M1 phenotype, although direct demonstration of this axis on TNT surfaces is still limited (Sun S. J. et al., 2013; Fu et al., 2023). Remarkably, precise tuning of nanotube spacing and diameter can direct macrophages toward an anti-inflammatory M2 phenotype, promoting secretion of IL-10, TGF-β, and other osteogenesis-favorable factors (Necula et al., 2022; Sun S. J. et al., 2013; Lü et al., 2015). The spacing between TNTs has been shown to significantly influence macrophage-like cell morphology and inflammatory responses (Necula et al., 2022). Recent studies have revealed that TNT-induced mitochondrial quality control plays a pivotal role in macrophage polarization, with decorin-mediated mitophagy and ROS regulation modulating NLRP3 inflammasome activation (Zhao et al., 2025). Concurrently, conformational changes in fibronectin within nanotopographical confines regulate inflammatory gene expression in macrophages through integrin α5β1 signaling (Qi et al., 2021). The RAW 264.7 macrophage response to TiO_2_ nanotube layers and the modulation of bone morphogenetic protein expression further corroborate this immune-osteogenic coupling phenomenon (Neacsu et al., 2014).

The early immune microenvironment orchestrated by TNT surfaces directly impacts subsequent infection risk and bone healing outcomes. When the immune response is dominated by the M2 phenotype, secreted bone morphogenetic protein (BMP)-2 and vascular endothelial growth factor (VEGF) synergistically promote BMSC osteogenic differentiation and angiogenesis (Bai et al., 2019; Wang et al., 2022). Conversely, a chronic M1-dominant inflammatory environment can suppress osteogenic activity through elevated TNF-α and other pro-inflammatory mediators, while generating ROS that may promote osteoblast apoptosis and create an inflammatory protein matrix conducive to bacterial adhesion (Abdulhameed et al., 2022; Fu et al., 2023). Long non-coding RNA (lncRNA) expression profiles in blood clots formed on TNT surfaces are closely related to surface topography and may regulate early bone healing through epigenetic mechanisms (Bai et al., 2021a).

In animal models, TNT surfaces significantly modulated the immune cell composition at the bone-implant interface during the early implantation period (1–4 weeks), reducing pro-inflammatory macrophage numbers and promoting early osteoid formation (Yun et al., 2013; Bai et al., 2021b). Magnesium-doped nanotube surfaces have been shown to promote M2 polarization via activation of the PI3K/Akt pathway in macrophages, thereby synergistically enhancing osseointegration (Qiao et al., 2020). Together, these findings establish the protein corona-immune microenvironment axis as the primary gateway of infection-osteogenesis crosstalk, with important implications for the clinical translation of immunomodulatory TNT surface optimization.

## 3 Molecular mechanisms of infection-osteogenesis crosstalk

The immune–osteogenic connections discussed below differ markedly in the strength of causal evidence, and we explicitly grade them to avoid overstating conclusions. Level I (well-established on TNT surfaces): TLR–NF-κB-mediated suppression of Runx2 and M1→M2 phenotypic switching, which have been causally validated in TNT-specific experiments. Level II (mechanistically plausible, extrapolated from other titanium surfaces or general osteoimmunology): the RANKL/OPG axis and HIF-1α-dependent metabolic reprogramming, which are supported by strong evidence in bone biology but lack dedicated TNT perturbation studies. Level III (correlative or inferred): neutrophil NETosis and T cell subset regulation, which rest mainly on temporal associations or findings from non-TNT biomaterials. Level IV (hypothetical): mechanisms proposed for future research, such as enzyme-responsive modulation of immune–bone coupling. Throughout this review we use precise terminology and flag each mechanism with its evidence level, so that readers can distinguish TNT-specific findings from extrapolations. These distinctions also identify priority areas for future mechanistic inquiry. Finally, we constructed Immunomodulation and Signaling Pathways on TiO2 Nanotube Surfaces (Figure 2).

**FIGURE 2:**
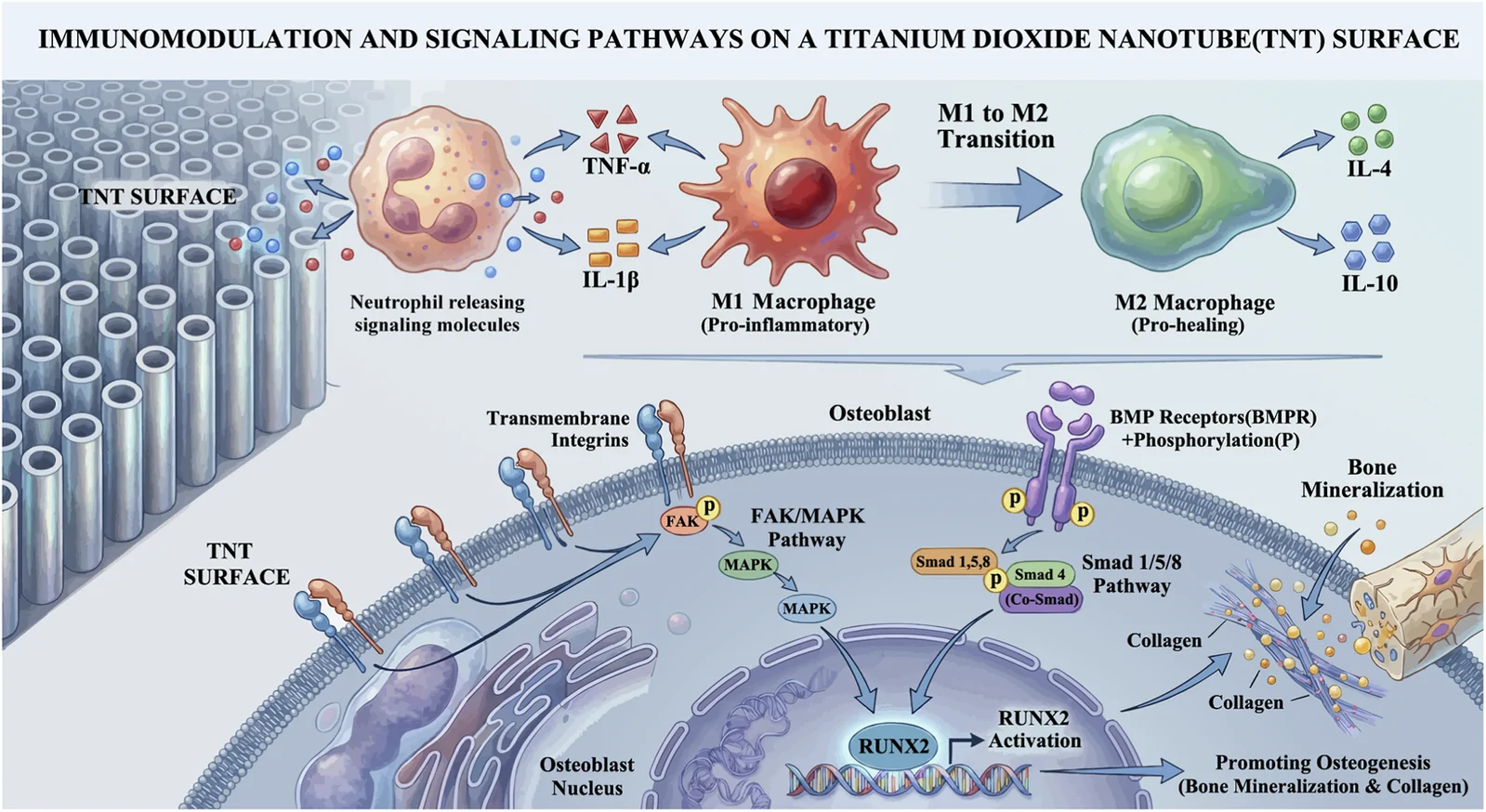
Immunomodulation and Signaling Pathways on TiO_2_ Nanotube Surfaces. Schematic overview of the molecular mechanisms underlying infection-osteogenesis crosstalk. Upper panel: Early immune response involving neutrophil recruitment and macrophage polarization transition from pro-inflammatory M1 phenotype (secreting TNF-α, IL-1β) to pro-healing M2 phenotype (secreting IL-4, IL-10)—a transition supported by direct evidence on TNT surfaces. Lower panel: Osteoblast signaling pathways including (i) integrin-mediated FAK/MAPK cascade and (ii) BMP receptor-activated Smad 1/5/8 pathway, both converging on RUNX2 activation.

### 3.1 Osteogenic suppression pathways induced by bacterial colonization and biofilm formation

Implant-associated infection is pathologically driven by bacterial colonization, proliferation, and biofilm formation on the implant surface. *S. aureus* is the most prevalent pathogen in orthopedic implant infections, followed by *S. epidermidis* and *Pseudomonas aeruginosa* (Li et al., 2019c; Wang et al., 2024). Once attached to TNT surfaces, bacteria rapidly secrete an extracellular polysaccharide matrix to form biofilms, which confer high tolerance to antibiotics and host immune defenses while continuously releasing soluble virulence factors that disrupt surrounding bone tissue (Xuan et al., 2023; Ma et al., 2024).

The signaling network through which bacterial products suppress osteogenesis is centered on the Toll-like receptor (TLR) pathway. In general osteoimmunology, lipoteichoic acid (LTA) from *S. aureus* and peptidoglycan (PGN) are recognized by TLR2 and TLR4, respectively, activating downstream MyD88-dependent NF-κB and MAPK signaling cascades; on TNT surfaces, NF-κB signaling has been implicated in the anti-inflammatory and osteogenic effects of bioactive coatings (Yang et al., 2021; Xuan et al., 2023), although the specific engagement of TLR2/TLR4 by bacterial products has not been directly demonstrated. Activated NF-κB transcriptionally upregulates pro-inflammatory cytokines such as TNF-α, IL-1β, and IL-6 and has been proposed to compete with the master osteogenic transcription factor Runx2 for transcriptional coactivators, thereby suppressing Runx2 activity; this NF-κB–Runx2 interplay is established in general osteoimmunology, but direct TNT-specific evidence is currently lacking. Resveratrol-loaded TNT coatings have been shown to attenuate inflammation and promote osteogenesis by inhibiting the NF-κB pathway (Yang et al., 2021). The sustained activation of MAPK pathways—particularly the p38 and JNK branches—has been shown to induce osteoblast apoptosis in the presence of inflammatory cytokines (Lu et al., 2020), compounding the impairment of bone repair capacity. Under stimulation by LPS and other Gram-negative bacterial products, TLR4 signaling has been shown to suppress the canonical Wnt/β-catenin pathway (Pei et al., 2017) and to upregulate sclerostin expression in osteocyte-like cells and macrophages (Sakamoto et al., 2019; Kwok et al., 2025), thereby antagonizing osteogenic signaling; however, this has not yet been directly examined on TNT surfaces.

Regulation of the RANKL/RANK/OPG axis represents another critical node in infection-osteogenesis crosstalk. Bacterial products stimulate osteoblasts and activated T cells to upregulate RANKL expression while suppressing osteoprotegerin (OPG) secretion, resulting in a significantly elevated RANKL/OPG ratio; this axis is firmly established in the general osteoimmunology literature on bacteria-induced bone loss, though TNT-specific perturbation experiments are lacking (Arciola et al., 2018). A high RANKL/OPG ratio activates RANK receptors on osteoclast precursors, promoting osteoclast maturation and bone resorptive activity, leading to peri-implant osteolysis. This mechanism is of central importance in both aseptic and infectious loosening of implants. Furthermore, bacterial LPS can directly act on osteoclast precursors through the TLR4-MyD88-IRAK4 pathway to enhance osteoclast survival and fusion capacity, a pathway demonstrated in general bone biology, though not yet examined on TNT surfaces.

Hypoxia within the biofilm microenvironment constitutes a further proposed mechanism of osteogenic suppression. Bacterial metabolism within the biofilm consumes substantial oxygen while the biofilm matrix impedes oxygen diffusion, creating a local hypoxic environment that may stabilize HIF-1α (Meng et al., 2022). Although moderate HIF-1α signaling has a pro-angiogenic role in normal bone repair, infection-associated sustained/excessive HIF-1α activation has been proposed to redirect osteoblasts toward glycolytic metabolism and inhibit mitochondrial oxidative phosphorylation, thereby impairing osteogenic mineralization; however, direct TNT-specific evidence for this metabolic switch remains limited. Recent studies indicate that antibacterial drug-loaded TNTs can restore normal spatiotemporal HIF-1α expression patterns by eliminating biofilms and improving local oxygen tension, providing a new molecular target for synergistic antibacterial-osteogenic therapy (Guan et al., 2025; Meng et al., 2022).

A methodological caveat deserves explicit attention when interpreting the antibacterial–osteogenic evidence reviewed above. Studies differ substantially in their infection models, and these differences directly affect conclusions about therapeutic efficacy. First, many antibacterial assays test planktonic bacteria, whereas mature biofilms—the clinically dominant form of implant-associated infection—are orders of magnitude more tolerant to antibiotics, ions, and host defenses; an agent that is bactericidal in suspension may show only bacteriostatic or anti-adhesive activity against established biofilms (Arciola et al., 2018; Busscher et al., 2012). Second, most studies use mono-species cultures (typically *S. aureus*), whereas clinical implant infections are frequently polymicrobial, and interspecies interactions can alter both biofilm architecture and antimicrobial susceptibility. Third, sterile osseointegration models do not recapitulate the infected-implant scenario, in which the immune milieu is fundamentally altered; reductions in bacterial counts should not be conflated with true treatment of an established infection, which requires eradication of biofilm-resident bacteria *in vivo*. Fourth, the route and timing of bacterial inoculation (peri-operative contamination vs. haematogenous seeding) influence whether a coating must prevent initial colonization or resolve a pre-existing infection. These distinctions should guide the design and interpretation of future studies and are summarized in Table 1.

**TABLE 1:** Comparison of major dual-functional TNT coating strategies by geometry and payload, antibacterial model and endpoint, osteogenic endpoint, *in vivo*/infection model, limitations, and translational status.

Strategy	Geometry & payload	Antibacterial model & endpoint	Osteogenic endpoint	*In Vivo*/Infection model	Limitations	Translational status
Ag-doped TNTs	TNT (20–30 nm); Ag NPs via sputtering/deposition; sustained Ag^+^ release	*S. aureus*/*E. coli*; planktonic and biofilm; CFU reduction and inhibition zones; bactericidal	MC3T3-E1/MSCs; ALP, Runx2, mineralization; dose-dependent (toxic >20 ppm Ag^+^)	Rat/rabbit osseointegration models; no infected-implant model	Narrow therapeutic window; long-term Ag^+^ toxicity; bio-corrosion	Pre-clinical only; no human trials (Gao et al., 2014; Yao et al., 2025b)
Cu/Zn-doped TNTs	TNT + Cu^2+^/Zn^2+^ doping; ROS/ion release	*S. aureus*; antifouling and planktonic assays; Cu requires UVA for efficacy	Zn: osteogenic via macrophage immunomodulation; Cu: angiogenesis coupling	Limited; mainly *in vitro*; few animal studies	Cu efficacy light-dependent; ion release kinetics poorly controlled	Pre-clinical; dose optimization needed (Jiang et al., 2020; Chen et al., 2020)
AMP-loaded TNTs	TNT + HHC-36/PGLa; sustained peptide release	*S. aureus*; planktonic and biofilm; membrane-lytic; bacteriostatic-to-bactericidal	Osteoblast viability maintained; degradation products non-toxic	Few *in vivo*; percutaneous implant model (HHC-36)	Peptide stability/degradation; production cost	Early pre-clinical (Miao et al., 2021; Xuan et al., 2023)
HA/bioactive glass composites	TNT + HA nanorods or bioglass; drug co-loading	Carrier-mediated (e.g., ciprofloxacin); planktonic assays	Osteoconductive; ALP & mineralization enhanced	Several rabbit/dog osseointegration models; no infection model	Coating adhesion; burst release; complex fabrication	HA clinically used; TNT-HA combo pre-clinical (Asadi et al., 2022; Lim et al., 2018)
Organic-inorganic coatings	TNT + chitosan/PDA/gelatin; drug-loaded mesh	Inherent + drug-carrier antibacterial; planktonic and early biofilm	Osteogenic via bioactivity and growth factors (BMP-2)	Rodent osseointegration models	Multi-step fabrication; batch variability; long-term degradation unknown	Pre-clinical (Kandel et al., 2021; Shaygani et al., 2023)
Smart responsive surfaces	pH/photo/enzyme-responsive TNT; on-demand release	pH-triggered Ag release (Dong et al., 2017); photothermal (Guan et al., 2025); photoelectron therapy (Yao et al., 2025a)	Early-stage; osteogenic response not fully characterized	Proof-of-concept small-animal biofilm-prevention models	Most conceptually advanced but least validated; activation reliability unclear	Early pre-clinical (Dong et al., 2017; Yao et al., 2025a)

Abbreviations: Ag, silver; Cu, copper; Zn, zinc; AMP, antimicrobial peptide; HA, hydroxyapatite; PDA, polydopamine; *S. aureus*, *Staphylococcus aureus*; S. epidermidis, *Staphylococcus* epidermidis; *E. coli*, *Escherichia coli*; MIC, minimum inhibitory concentration; CFU, colony-forming unit; ALP, alkaline phosphatase; BIC, bone-implant contact.

### 3.2 Immune cell-mediated bidirectional regulation of infection-osteogenesis crosstalk

#### 3.2.1 Macrophages and the NLRP3 inflammasome

Immune cells—particularly macrophages, neutrophils, and T cells—play bidirectional regulatory roles in infection-osteogenesis crosstalk, functioning as the first line of host defense while simultaneously participating in bone metabolism regulation through their secreted cytokine and chemokine networks.

Macrophages are the most abundant immune cell population at the implant interface, and their polarization state in the infectious microenvironment dictates the direction of bone healing. When infection occurs on TNT surfaces, bacterial products (LTA, LPS) and damage-associated molecular patterns (DAMPs) strongly drive macrophages toward the classically activated M1 phenotype (Fu et al., 2023; Sun S. J. et al., 2013). M1 macrophages secrete copious amounts of TNF-α, IL-1β, IL-6, and iNOS/NO. While these factors eliminate invading bacteria, they simultaneously inhibit BMSC osteogenic differentiation through activation of NF-κB and STAT1 signaling pathways. Conditioned medium from M1 macrophages has been shown to significantly reduce ALP activity and mineralized nodule formation in BMSCs (Bai et al., 2019; Feng et al., 2020). Additionally, andrographolide-loaded TNT coatings have demonstrated the capacity to inhibit bacterial biofilm formation while concurrently enhancing osteogenic activity (Feng et al., 2020). However, failure of the M1 response to transition in a timely manner to a reparative M2-dominant phenotype results in chronic inflammation—the core reason why infectious bone defects around implants are difficult to heal.

The NLRP3 inflammasome has been proposed as a central hub in infection-osteogenesis crosstalk. Bacterial LPS primes NF-κB-dependent transcription of pro-IL-1β and NLRP3 (signal 1), while bacterial ATP, pore-forming toxins, and ROS trigger NLRP3 inflammasome assembly and caspase-1 activation (signal 2). Active caspase-1 cleaves pro-IL-1β and pro-IL-18 into their mature forms and induces pyroptosis—a canonical pathway well characterized in general inflammasome biology, although its specific activation on TNT surfaces awaits direct confirmation (Zhao et al., 2025). Active caspase-1 also cleaves Gasdermin D to form membrane pores that release IL-1β, amplifying local inflammation. IL-1β itself is a potent bone resorption-stimulating factor that promotes osteoclastogenesis by upregulating c-Fos and NFATc1 in osteoclasts, an effect documented in general bone biology (Liu et al., 2022). Crucially, TNT nanotopography has been shown to suppress NLRP3 inflammasome hyperactivation by reducing ROS production through modulation of mitochondrial quality control, a process involving decorin-mediated mitophagy (Zhao et al., 2025).

#### 3.2.2 Neutrophils and NETosis

Neutrophils are the first immune cells recruited to the implant surface during early infection. In general host defense, neutrophils can release neutrophil extracellular traps (NETs) through NETosis to capture and kill bacteria; however, direct demonstration of NETosis on TNT surfaces is currently lacking, and its role in implant-associated infection remains largely inferred from other biomaterial contexts (Arciola et al., 2018). NETs contain histones, matrix metalloproteinases (MMPs), and ROS that may exert dual effects on surrounding bone tissue: low-level NETs contribute to bacterial clearance and provide temporary scaffolds for bone remodeling, but excessive NET formation and impaired clearance have been associated with exacerbated osteolysis and delayed bone healing in other settings (Rahnamaee et al., 2023). Photoelectron therapy, which induces local electron transfer, has been demonstrated to reduce bacterial biofilm formation on TNT surfaces (Yao K. et al., 2025); whether it concurrently modulates the extent of neutrophil NETosis requires further investigation.

#### 3.2.3 T cell subsets

T cell subsets have been suggested to play significant roles in infection-osteogenesis crosstalk, although direct evidence on TNT surfaces remains sparse. Th17 cells, through IL-17 secretion, recruit neutrophils and activate RANKL-mediated osteoclastogenesis, exacerbating infection-associated bone resorption. Conversely, regulatory T cells (Tregs) suppress excessive inflammation and promote osteogenesis via IL-10 and TGF-β secretion—a paradigm established in general osteoimmunology (Qiao et al., 2020). In TNT surface functionalization, loading immunomodulatory factors such as IL-4 or BMP-2 has been shown to promote osseointegration in rodent models (Zhao et al., 2016; Liu et al., 2022). Bioactive glass composite coatings have also demonstrated enhanced osteoblast compatibility and antibacterial properties (Das et al., 2008). Furthermore, osteoimmune-modulating, BMP-2-eluting anodized 3D-printed titanium surfaces can modulate early immune cell recruitment, thereby improving implant osseointegration rates (Ali et al., 2024).

## 4 Synergistic design strategies for multifunctional TNT surfaces

### 4.1 Dual-functional antibacterial-osteogenic coating strategies

Building upon the mechanistic understanding of infection-osteogenesis crosstalk, substantial research efforts have been devoted to integrating antibacterial and osteogenic functions on the TNT platform, aiming to transcend the limitations of conventional monofunctional coatings through synergistic “kill two birds with one stone” strategies (Figure 3).

**FIGURE 3:**
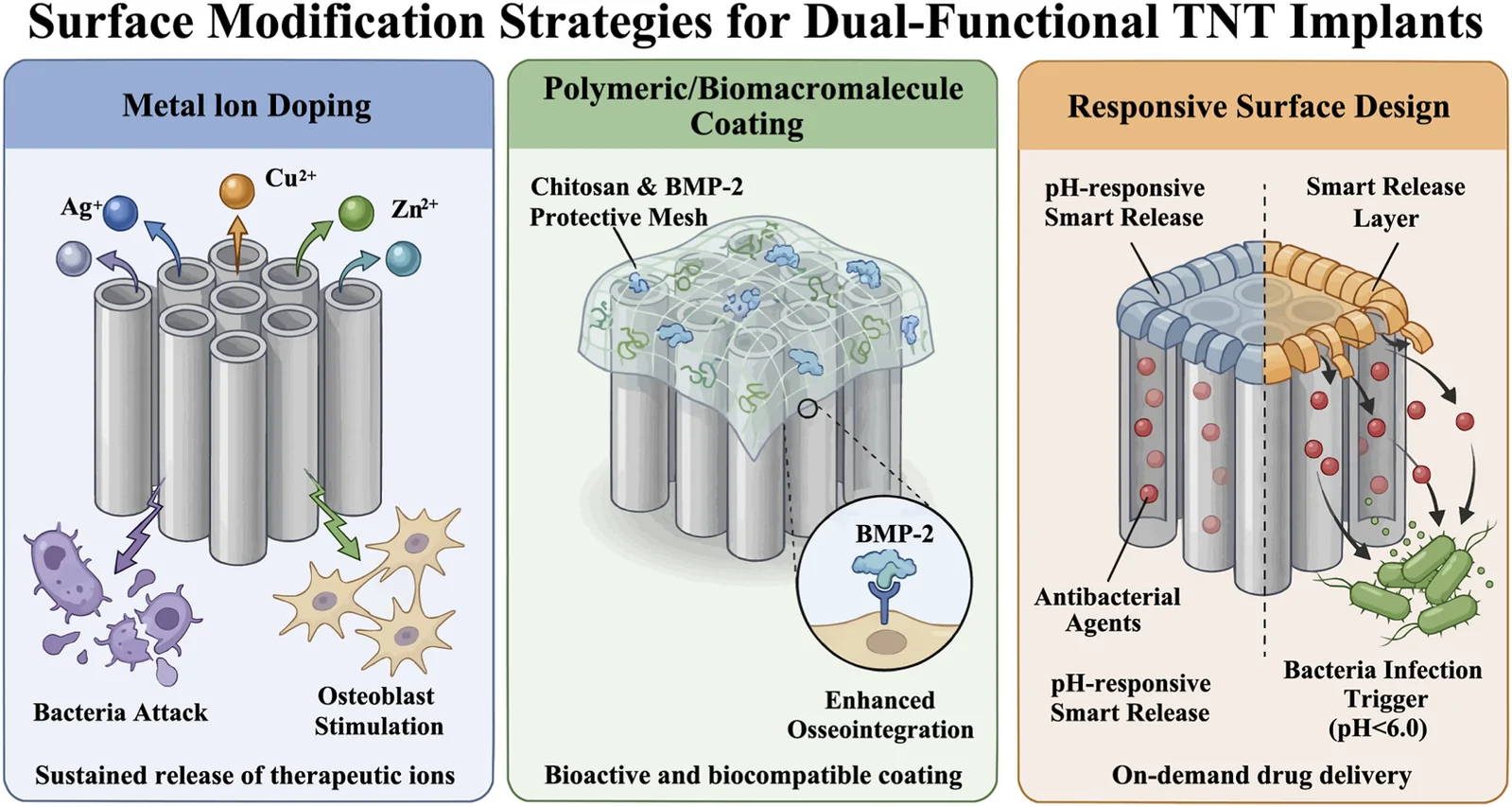
Surface Modification Strategies for Dual-Functional TNT Implants. Schematic summary of three major categories of TNT surface engineering approaches. Left: Metal ion doping (Ag^+^, Cu^2+^, Zn^2+^) enabling sustained release of therapeutic ions, where Ag^+^/Cu^2+^ exert antibacterial effects and Zn^2+^ promotes osteoblast stimulation. Center: Polymeric/biomacromolecule coating (chitosan/BMP-2 protective mesh) providing bioactive and biocompatible interfaces for enhanced osseointegration. Right: Responsive surface design with pH-responsive smart release mechanism, maintaining drug encapsulation under normal physiological conditions (pH 7.4) and triggering antibacterial agent release upon bacterial infection-induced acidification (pH < 6.0).

Metal ion doping represents one of the most straightforward approaches. Silver (Ag), with its broad-spectrum antibacterial activity and relatively low propensity for inducing bacterial resistance, has been extensively investigated. Ag nanoparticles have been loaded into TNT lumens and surfaces via magnetron sputtering, ion implantation, or electrochemical deposition, achieving localized Ag^+^ release that kills *S. aureus* and *E. coli* while inhibiting biofilm formation (Gao et al., 2014; Yao Y. et al., 2025). Hybrid silver/reduced graphene oxide-decorated TNTs have demonstrated enhanced antibacterial performance (Esfandiari et al., 2014). However, Ag^+^ concentration presents a double-edged sword—excessive levels (>20 ppm) exhibit significant cytotoxicity. Optimization strategies include leveraging the nanoconfinement effect of TNTs to achieve slow, sustained Ag^+^ release, controlling Ag nanoparticle size below 15 nm to reduce toxicity, and combining Ag^+^ with other pro-osteogenic ions (Lin et al., 2016; Xu et al., 2017). Optimized TNT coatings with silver nanostructures have been shown to maintain antibacterial activity and promote osseointegration in animal models for up to 4 weeks post-implantation (Uhm et al., 2016; Gao et al., 2023). Biocompatible nanostructured silver-incorporated surfaces have similarly demonstrated effective resistance against bacterial biofilms (Savargaonkar et al., 2023; Zhang et al., 2018).

Copper (Cu) and zinc (Zn), as essential trace elements in the human body, possess dual antibacterial and pro-osteogenic functions. Cu^2+^ exerts antibacterial effects through ROS generation while simultaneously supporting angiogenesis-osteogenesis coupling; copper-modified TNT surfaces have demonstrated antifouling and antibacterial activity (Jiang et al., 2020; Savargaonkar et al., 2023). Zn^2+^ promotes osteogenesis through immunomodulatory effects on macrophages while maintaining antibacterial activity (Chen et al., 2020). Zn-doped TNT surfaces have been shown to improve osteogenesis ability through influencing the immunomodulatory function of macrophages (Chen et al., 2020). Importantly, co-doping of Cu and Ag produces synergistic antibacterial effects, with enhanced photocatalytic activity from Cu_2_O/Ag heterojunctions further improving ROS generation efficiency (Esfandiari et al., 2014).

Despite the promising results described above, it is important to acknowledge that metal-ion doping strategies are not universally successful. Several studies have reported a narrow or absent therapeutic window for Ag- and Cu-doped TNT coatings. For example, The antibacterial efficacy of ZnO and Ag nanoparticles delivered via TNT composite layers plateaued at sub-therapeutic doses, while higher concentrations induced cytotoxicity (Roguska et al., 2015). Similarly, Copper-doped TiO_2_ nanotube coatings showed dose-dependent antibacterial activity only under specific UVA illumination conditions (Oder et al., 2020). Silver nanoparticle size and distribution critically determine the balance between bactericidal activity and cytotoxicity (Esfandiari et al., 2014). These findings underscore the need for precise control over ion release kinetics and the importance of evaluating doping strategies under physiologically relevant conditions. Moreover, to date, no metal-ion-doped TNT coating has advanced to clinical trials, and the existing evidence is derived exclusively from *in vitro* and small-animal studies. This fundamental limitation should be carefully considered when assessing the translational potential of these approaches.

Beyond metal ions, non-metal elemental doping has also emerged as a promising strategy. Yang et al. demonstrated that iodine-doped TiO_2_ nanotube coatings (I-TiO_2_), fabricated by anodization followed by electrophoretic deposition of iodine, exhibit significantly enhanced antimicrobial activity against *Staphylococcus aureus* compared to undoped TNTs, providing a novel elemental approach to preventing implant-associated infections (Yang et al., 2023).

Antimicrobial peptides (AMPs) and bioactive molecules represent another highly promising strategy. Unlike conventional antibiotics, AMPs rapidly kill bacteria through membrane-lytic mechanisms and are less prone to inducing resistance. AMPs such as HHC-36 and PGLa have been successfully loaded into TNTs, achieving sustained release and excellent antibacterial activity (Miao et al., 2021; Xuan et al., 2023). Importantly, the degradation products of AMPs are amino acids that are non-toxic to osteoblasts, and certain AMPs (e.g., PGLa) also exhibit anti-inflammatory activity that may support a pro-healing microenvironment (Xuan et al., 2023). Nitric oxide (NO), an endogenous gaseous signaling molecule, promotes bone formation at low concentrations and exerts potent antibacterial activity at high concentrations. Modification of TNT surfaces with copper-catalyzed endogenous NO release has been achieved via polydopamine-based coatings, enabling on-demand infection clearance and improvement of the vascular microenvironment (Jiang et al., 2020). Honokiol-loaded TNT drug delivery systems have similarly demonstrated combined anti-inflammatory and pro-osteogenic effects (Tang et al., 2023).

Organic-inorganic composite coatings integrate the inorganic TNT substrate with bioactive organic components such as chitosan, hyaluronic acid, gelatin, and polydopamine, achieving multifaceted antibacterial, anti-inflammatory, and osteogenic synergy (Li H. et al., 2019; Kandel et al., 2021). Chitosan coatings possess intrinsic antibacterial properties while serving as drug release barriers and cell adhesion matrices; chitosan-coated TNTs have been shown to improve drug (e.g., metformin) release and osteoblast differentiation (Hashemi et al., 2020). Chitosan-crosslinked polyaniline-patterned TNT surfaces have shown biomimetic regulation of osteoblast behavior (Kandel et al., 2021). Nanofibrillated chitosan-coated highly ordered TNT/graphene composites have further enhanced mechanical properties and osteoinductivity (Rahnamaee et al., 2021). Polydopamine (PDA) coatings adhere firmly to TNT surfaces, providing abundant catechol functional groups for subsequent grafting of AMPs, BMP-2, or osteoinductive calcium-phosphate compounds (Shaygani et al., 2023). Calcium peroxide (CaO_2_)-incorporated PDA/TNT coatings enable sustained release of Ca^2+^ and O_2_, achieving synergistic antibacterial and pro-osteogenic effects on BMSC osteogenic differentiation (Shaygani et al., 2023).

Nano-hydroxyapatite (HA) and bioactive glass composite coatings are designed to mimic the inorganic composition of natural bone, providing osteoconductivity and neutralizing the local acidic environment. After electrodeposition of HA nanorods on TNT surfaces, antibacterial drugs (e.g., ciprofloxacin) can be co-loaded into both the HA layer and the TNT lumens to create a dual-layer drug delivery system: the HA layer provides rapid release for early antibacterial action, while the TNT lumens sustain long-term release for prophylaxis (Asadi et al., 2022; Shaygani et al., 2023). Magnetron-sputtered tantalum-coated TNTs have also demonstrated sustained drug release and osteoinductive performance (Zhan et al., 2024). Silver-substituted fluorhydroxyapatite-TiO_2_ composite coatings have achieved efficient killing of multiple clinically isolated strains while maintaining good osteogenic activity (Huang et al., 2018). Furthermore, Ca-P/Ag/TiO_2_ nanotube composite coatings prepared by electrochemical deposition have demonstrated synergistic antibacterial and osteoconductive effects (Roguska et al., 2012). Hydroxyapatite coatings directly deposited on TiO_2_ nanotube arrays via the sol-gel method have also been validated for enhanced implant performance (Lim et al., 2018; Li et al., 2019b).

Temporal drug release strategies are central to synergistic design. An ideal TNT coating should satisfy the temporal sequence of “early high-concentration antibacterial → mid-term immunomodulation → late sustained osteogenesis.” Current approaches to achieve this include: (i) utilizing the dual-layer structure of TNT lumens and surface coatings for sequential release (Shaygani et al., 2023); and (ii) leveraging the nanoconfinement effect of nanotubes and surface functional groups (e.g., PDA) interactions to modulate release kinetics (Zhan et al., 2024). Icariin/TiO_2_ nanotube composite coatings and strontium/icariin co-loaded coatings have demonstrated temporal release and synergistic osteogenic effects in both *in vitro* and *in vivo* experiments (Zhu et al., 2021). The comparison of major dual-functional strategies is summarized in Table 1.

### 4.2 Smart responsive surfaces and future perspectives

Despite the significant progress achieved with dual-functional TNT coatings, fixed release patterns continue to face challenges related to “over-/under-antimicrobial” effects and inter-individual variability. In recent years, smart responsive TNT surfaces have attracted growing interest as a next-generation design paradigm, and flexible multifunctional TNT platforms have been developed for diverse biological interfacing applications (Amani Hamedani et al., 2024).

pH-responsive surfaces exploit the local acidity of the infectious microenvironment (pH 5.0–6.5) compared to normal tissue (pH 7.4). By coating TNT surfaces with pH-sensitive polymers or responsive nanocarriers, antibacterial agents can be released preferentially under acidic infectious conditions while remaining in a “dormant” state at physiological pH; for example, pH-dependent silver-releasing titanium implants have been developed to control peri-implant infection (Dong et al., 2017). This “on-demand drug release” mode minimizes unnecessary toxicity to normal tissue cells. Superhydrophobic TNT arrays have also demonstrated potential for reduced bacterial adhesion and easy clearance (Bartlet et al., 2018).

Photothermal/photocatalytic responsive surfaces leverage the inherent photocatalytic properties of TiO_2_ or the photothermal conversion effect of near-infrared (NIR)-absorbing nanomaterials (e.g., MXene) for photothermal sterilization. Under NIR irradiation, MXene-based composite coatings can generate localized heating to eliminate bacterial biofilms within a short time frame while supporting osteogenic responses (Guan et al., 2025). Visible-light-triggered drug release systems have also been developed for TiO_2_ nanotube arrays, enabling controlled antibacterial therapy (Xu et al., 2016b). Photoelectron therapy—which utilizes photogenerated electron transfer from TiO_2_ under ultraviolet/visible light to disrupt the bacterial respiratory chain—has been validated in small animal models for effective prevention of biofilm formation on TNT implants (Yao K. et al., 2025). Graphitic carbon nitride (g-C_3_N_4_)-sensitized TNTs extend photocatalytic activity into the visible light region, enabling efficient antibacterial effects without ultraviolet irradiation (Xu et al., 2016a).

Enzyme-responsive surfaces represent a conceptually attractive direction that exploits the aberrant expression of specific enzymes (e.g., bacterial proteases, phospholipases, hyaluronidases) in the infectious microenvironment, employing enzyme-degradable TNT surface coatings. When bacterial enzymes cleave the coating, encapsulated antibacterial agents or osteogenic factors would be released. This strategy could enable specific responses to bacterial populations without disturbing commensal flora. It should be emphasized, however, that enzyme-responsive TNT systems remain at the conceptual stage, and no dedicated experimental validation on TNT surfaces has yet been reported.

Immunomodulatory coatings represent the most cutting-edge design direction. Rather than directly killing bacteria or passively releasing osteogenic factors, immunomodulatory coatings aim to actively shape the immune microenvironment at the implant interface, harnessing the host own immune system to coordinate antibacterial and bone repair processes. This can be achieved through TNT surface topographical optimization to directly regulate macrophage phenotype (Sun S. J. et al., 2013; Necula et al., 2022), or by loading immunomodulatory factors (e.g., IL-4, IL-10, BMP-2) to guide the M1→M2 transition, which has been demonstrated to improve osseointegration in rodent models (Zhao et al., 2016; Liu et al., 2022). The decorin/TNT platform, which suppresses NLRP3 hyperactivation through modulation of mitochondrial quality control, represents a promising breakthrough in the immuno-metabolic regulation direction (Zhao et al., 2025). Similarly, titania implants engineered for glypican-mediated regulation of BMP-2 bioactivity illustrate how surface-immobilized biomolecules can steer osteogenic signaling (Bariana et al., 2018).

Clinical translation challenges remain the core bottleneck for smart TNT surfaces. Major obstacles include: (1) batch-to-batch consistency and fabrication reproducibility in large-scale TNT production; (2) unclear coating durability, mechanical fatigue, and degradation behavior of nanotubes during long-term *in vivo* implantation; (3) sterilization compatibility and shelf-life stability of multi-component coatings; (4) potential toxicity of released ions or drugs, release kinetics, and systemic exposure; (5) potential discrepancies in therapeutic efficacy assessment due to immunological differences between animal models and human clinical settings; and (6) complex biosafety and regulatory approval pathways for multi-component smart coatings (Busscher et al., 2012). Moreover, current models of infection-osteogenesis crosstalk on TNT surfaces are predominantly based on *in vitro* two-dimensional co-culture systems that lack the three-dimensional immune-bone microenvironment fidelity achievable *in vivo*. The development of microfluidic bone-on-a-chip platforms and 3D-bioprinted immune-bone organoid models holds promise for addressing this gap (Wang et al., 2022). Additionally, the *in situ* construction of TiO_2_ nanotubes on 3D-printed titanium alloy surfaces is emerging as a new direction for customized implants (Ali et al., 2024).

Looking forward, the integration of artificial intelligence-assisted materials genomics, high-throughput TNT surface array screening, and single-cell multi-omics analysis technologies holds the potential to accelerate the discovery of optimal immune-osteogenic synergistic surface parameters. Whole-genome expression analysis by RNA sequencing has provided systematic tools for deciphering the molecular regulatory network of TiO_2_ nanotube-mediated osteoblast differentiation (Peng et al., 2010), and future efforts should further strengthen the deep integration of genomics, proteomics, and materials science. Translating the concept of infection-osteogenesis crosstalk from a scientific framework into clinically actionable surface design principles will require sustained interdisciplinary collaboration.

## 5 Conclusion

TiO_2_ nanotube surfaces, with their unique nanotopographical features and excellent potential for functional modification, provide an ideal platform for addressing the dual clinical challenges of implant-associated infection and insufficient osseointegration. This review has systematically examined the biological mechanisms of infection-osteogenesis crosstalk on TNT surfaces and the synergistic design strategies developed to harness this crosstalk.

TNT morphological parameters—particularly nanotube diameter—constitute the physical foundation for differentially regulating osteoblast behavior and bacterial responses. TNT morphological parameters exert a phase-dependent regulatory effect: smaller diameters (∼20–30 nm) are optimal for reducing bacterial adhesion and supporting early cell anchorage, whereas larger diameters (∼60–80 nm) are more effective in driving osteogenic differentiation. This divergence highlights the need to define the “optimal” diameter in relation to the specific therapeutic goal, and motivates future designs—such as gradient or hybrid architectures—to achieve spatiotemporal synergy across different healing stages. The protein corona-mediated early immune microenvironment serves as a critical “gatekeeper” determining infection risk and bone healing trajectory.

The molecular mechanisms of infection-osteogenesis crosstalk involve direct suppression of osteogenic differentiation by bacterial virulence factors through the TLR-NF-κB pathway, RANKL/OPG imbalance-driven osteoclast activation, and HIF-1α-dependent bone metabolic reprogramming in the biofilm hypoxic microenvironment. Within the immune cell network, macrophage M1/M2 polarization imbalance, NLRP3 inflammasome hyperactivation, and the double-edged sword effect of NETs represent key immunomodulatory nodes.

The strength of evidence is uneven across the pathways discussed. The TLR-NF-κB and RANKL/OPG axes, as well as macrophage M1/M2 polarization, are supported by consistent, multi-laboratory evidence on TNT surfaces and can be considered well-established paradigms. In contrast, other mechanisms—particularly HIF-1α-mediated metabolic reprogramming, neutrophil NETosis, and T cell subset regulation—rest on limited or indirect evidence, with few TNT-specific studies and minimal *in vivo* validation. Moreover, the literature contains notable inconsistencies that warrant explicit acknowledgment: the reported “optimal” TNT diameter varies depending on whether the endpoint is cell adhesion (15–30 nm) or osteogenic differentiation (60–80 nm); HIF-1α exerts opposing effects on bone repair depending on the duration and intensity of hypoxic signaling, yet the threshold on TNT surfaces remains undefined; and NETosis has been described as both protective and pathological in bone healing contexts, with its TNT-specific modulation entirely unexplored. These gaps and contradictions do not invalidate the crosstalk concept but underscore the need for more rigorous, direct, and comparative investigations—particularly studies that systematically evaluate multiple mechanisms within the same experimental framework—before the proposed pathways can be confidently translated into design principles. We have highlighted these limitations throughout the review to provide readers with a balanced perspective on the current state of the field.

Synergistic design strategies have evolved from simple functional superposition toward multifunctional integration and intelligent responsiveness. Metal ion doping, antimicrobial peptide loading, organic-inorganic composite coatings, and temporal drug release systems have demonstrated promising synergistic antibacterial-osteogenic effects. pH-responsive, photothermal/photocatalytic, enzyme-responsive, and immunomodulatory coatings represent the next-generation of smart surfaces with the potential for “on-demand antibacterial and precision osteogenic” therapy.

Five priority research questions emerge from the gaps identified above: ① What are the causal contributions of NETosis and T cell subsets to TNT-mediated bone healing outcomes, and can their modulation extend beyond the current correlative evidence base? ② Where is the threshold between beneficial and deleterious HIF-1α signaling on TNT surfaces under biofilm-associated chronic hypoxia, and can it be therapeutically targeted without compromising early revascularization? ③ Can the conflicting diameter requirements for bacterial exclusion (∼20–30 nm) versus osteogenic differentiation (∼60–80 nm) be reconciled through hierarchical, gradient, or temporally dynamic TNT architectures? ④ Which combination of metal ions, peptides, or immunomodulatory agents achieves the widest therapeutic window in large-animal models that recapitulate human bone quality and immune responses? ⑤ What are the critical safety, pharmacokinetic, and batch-to-batch consistency data required to progress the most promising TNT-based system into a first-in-human trial?
